# Cell-free DNA analysis in healthy individuals by next-generation sequencing: a proof of concept and technical validation study

**DOI:** 10.1038/s41419-019-1770-3

**Published:** 2019-07-11

**Authors:** Ilaria Alborelli, Daniele Generali, Philip Jermann, Maria Rosa Cappelletti, Giuseppina Ferrero, Bruna Scaggiante, Marina Bortul, Fabrizio Zanconati, Stefan Nicolet, Jasmin Haegele, Lukas Bubendorf, Nicola Aceto, Maurizio Scaltriti, Giuseppe Mucci, Luca Quagliata, Giuseppe Novelli

**Affiliations:** 1 Institute of Pathology, University Hospital Basel, 4031 Basel, Switzerland; 2 Breast Cancer Unit and Translational Research Unit, ASST Cremona, Viale Concordia 1, 26100 Cremona, Italy; 3 Department of Medical Surgery and Health Sciences, University of Trieste, 34129 Trieste, Italy; 4 Department of Life Sciences, University of Trieste, Via Giorgeri, 1, 34127 Trieste, Italy; 5 Cancer Metastasis Laboratory, Department of Biomedicine, University of Basel, 4058 Basel, Switzerland; 6 Human Oncology & Pathogenesis Program (HOPP), Memorial Sloan Kettering Cancer Center, 1275 York Avenue, New York, NY 10065 USA; 7 Department of Pathology, Memorial Sloan Kettering Cancer Center, 1275 York Avenue, 10065 New York, NY USA; 8 Bioscience Institute, Via Rovereta 42, Falciano, 47891 San Marino, Italy; 9 Department of Biomedicine and Prevention, University of Rome Tor Vergata, Rome, Italy; 10 IRCCS Neuromed, Pozzilli, Italy; 11 Present Address: Novartis Institutes for BioMedical Research, 4056 Basel, Switzerland; 12 Present Address: Thermo Fisher Scientific, 6300 Zug, Switzerland

**Keywords:** Diagnostic markers, Cancer genomics

## Abstract

Pre-symptomatic screening of genetic alterations might help identify subpopulations of individuals that could enter into early access prevention programs. Since liquid biopsy is minimally invasive it can be used for longitudinal studies in healthy volunteers to monitor events of progression from normal tissue to pre-cancerous and cancerous condition. Yet, cell-free DNA (cfDNA) analysis in healthy individuals comes with substantial challenges such as the lack of large cohort studies addressing the impact of mutations in healthy individuals or the low abundance of cfDNA in plasma. In this study, we aimed to investigate the technical feasibility of cfDNA analysis in a collection of 114 clinically healthy individuals. We first addressed the impact of pre-analytical factors such as cfDNA yield and quality on sequencing performance and compared healthy to cancer donor samples. We then confirmed the validity of our testing strategy by evaluating the mutational status concordance in matched tissue and plasma specimens collected from cancer patients. Finally, we screened our group of healthy donors for genetic alterations, comparing individuals who did not develop any tumor to patients who developed either a benign neoplasm or cancer during 1–10 years of follow-up time. To conclude, we have established a rapid and reliable liquid biopsy workflow that allowed us to study genomic alterations with a limit of detection as low as 0.08% of variant allelic frequency in healthy individuals. We detected pathogenic cancer mutations in four healthy donors that later developed a benign neoplasm or invasive breast cancer up to 10 years after blood collection. Even though larger prospective studies are needed to address the specificity and sensitivity of liquid biopsy as a clinical tool for early cancer detection, systematic screening of healthy individuals will help understanding early events of tumor formation.

## Introduction

Genomic instability arises in normal cells through accumulation of genetic and epigenetic changes and has been shown to occur over a variable time span, ranging from years to decades^1–4^. The vast majority of normal cells that have acquired mutations is cleared away by the immune system, while a minimal fraction might eventually progress and give rise to cancer^5^. Upon development of cancer, specific genomic alterations can be identified and used to provide the rationale for specific treatment options. Monitoring genomic changes could hence be crucial to identify early mutational events that are associated with higher risk of developing cancer, but it is largely unfeasible using traditional tissue-based approaches due to the lack of observable tumor lesions. In contrast, liquid biopsy might offer the possibility of detecting early genomic aberrations and investigating cancer evolution in a minimally invasive fashion. Liquid biopsy is a broad term that refers to testing body fluids such as blood or urine for biomarkers reconcilable with a medical condition. In the field of oncology, liquid biopsy mainly pertains to the analysis of circulating tumor DNA (ctDNA) in blood. Circulating tumor DNA represents only a minor fraction (<0.1–10%) of the total circulating cell-free DNA (cfDNA)^6^, which is derived by cell death associated to physiological tissue remodeling events^7^. The majority of DNA fragments found in the circulation measures ~180 nucleotides in size^8^, suggesting that apoptosis and necrosis are responsible for cfDNA shedding. Interestingly, the blood of cancer patients typically presents higher levels of circulating cfDNA compared to healthy individuals^9,10^.

A growing body of evidence supports ctDNA-based analysis of cancer-associated hotspot mutations as a cost-effective and highly sensitive tool, complementary to tissue molecular profiling^11–15^. In clinical settings, ctDNA analysis has been applied to monitor response to treatment, to detect residual disease and to identify mechanisms of resistance to therapy^16–18^. Currently, the most common clinical use of liquid biopsy is the detection of resistance-associated mutations to inform treatment decision^19–22^. The introduction of molecular barcodes has considerably enhanced the sensitivity of sequencing methods at the price of additional costs linked to the high depth of sequencing required (i.e. ~25,000 coverage)^13,23,24^. Taking advantage of this and further technological developments, several studies have described clinically relevant genetic alterations in patients with early-stage cancers at a sensitivity below one mutant template molecules per milliliter of plasma^9,25–28^. Nevertheless, among the potential clinical applications of ctDNA analysis, early detection remains the most ambitious. Several challenges need to be addressed and large validation studies will be required to establish the sensitivity and specificity for such testing approach^29^. The presence of somatic mutations in asymptomatic patients, related to clonal hematopoiesis^28^ as well as clonal expansion in healthy tissue^30–33^, could potentially lead to false-positive calls. Moreover, the recovery and characterization of cfDNA in healthy individuals might prove challenging, given that cfDNA is less abundant in these subjects^34,35^ and only a few studies have reported the analysis of cfDNA in healthy controls^28,36^.

An adequate technical validation is therefore required to allow the implementation of liquid biopsy as a tool for early cancer detection and prove that extraction and analysis of cfDNA isolated from healthy individuals is technically achievable. To this end in our proof of principle study, we examined the feasibility cfDNA interrogation in a collection of 114 individuals that were clinically healthy (i.e. not affected by any manifest medical condition) at the time of blood draw.

First, we analyzed the impact of pre-analytical factors such as cfDNA yield and quality on sequencing performance parameters as molecular coverage and limit of detection, comparing samples from healthy and cancer donors.

We then evaluated the reliability of our testing strategy by analyzing cfDNA samples obtained from patients with a histologically confirmed diagnosis of breast or lung cancer. Specifically, we assessed the concordance of specific genetic alterations detected in matched tissue and plasma specimens. Finally, we investigated the mutational status of a group of healthy donors comprising both individuals that did not develop any tumor within 1 to 10 years (average = 8.5 years; Table 1) of follow-up as well as individuals that developed either a benign neoplasm or cancer during the follow-up time. Altogether, our study demonstrates the technical feasibility of extracting and analyzing cfDNA in healthy individuals to study genomic alterations, by means of molecular barcoded next-generation sequencing (NGS).

**Table 1:** Patient characteristics

Patient characteristics (*n* = 177)	*n* (%)
Age (years)	
Mean (SD)	63 (11)
Sex (*n*)	
Male	19 (11)
Female	157 (89)
Clinical status at blood collection (*n*)	
No tumor (healthy)	114 (64)
Breast cancer	9 (5)
Lung cancer	54 (31)
Clinical status at follow-up^a^ (*n*)	
No tumor (healthy)—Group I	25 (14)
Benign breast condition—Group II	52 (29)
Breast cancer—Group III	15 (8)
Other tumors—Group IV	14 (8)
Missing information (lost to follow-up)	8 (5)
Follow-up* time from blood collection (years)	
Mean (range)—All Groups	8.5 (1.1–10.1)
Mean (range)—Group I	8.7 (4.6–10.1)
Mean (range)—Group II	8.4 (1.1–10.0)
Mean (range)—Group III	9.0 (4.3–10.0)
Mean (range)—Group IV	8.4 (4.1–9.6)
Molecular analysis (*n*)	
Plasma—cfDNA extraction	177 (100)
Plasma—NGS analysis	93 (53)
Tissue—NGS analysis	38 (21)

^a^Follow-up data have been collected in the frame of this study only for donors that were clinically healthy at blood collection

## Materials and methods

### Patients

One hundred and fourteen healthy donors undergoing a control screening mammography test and nine breast cancer patients undergoing treatment at the Breast Cancer Unit and Translational Research Unit of the Hospital of Cremona (Italy) were selected for this study (Ethical approval protocol nr. Ex01/4111/04). In addition, 54 lung cancer patients undergoing treatment at the University Hospital Basel (Switzerland) were selected for this study (Ethical approval protocol EKBB/EKNZ 31/12). The study was performed in compliance with all relevant ethical regulations. More plasma was available in the cancer patient group (from 1.5 to 5.5 ml) in cancer patients because 2 × 10 ml of whole blood was collected for each patient. Conversely, only 1 × 10 ml whole blood was drawn from healthy individuals, part of which was used for other analyses, resulting in a final plasma volume ranging from 0.4 to 2.0 ml. Follow-up data have been collected in the frame of this study only for donors that were clinically healthy at blood collection.

### cfDNA/DNA extraction from plasma and tissue samples

Blood samples were collected in either K_2_EDTA tubes (BD Vacutainer® Blood Collection Tubes, Becton Dickinson, Franklin Lakes, USA) and Cell-Free DNA BCT® (Streck, La Vista, NE). The plasma fraction was separated from the blood cells by two consecutive rounds of centrifugation for 30 min at room temperature at 1600 × *g*. The collected plasma was aliquoted and stored at −80 °C until use. cfDNA was extracted from plasma volumes ranging from 0.4 to 5.5 ml using the MagMax Cell-Free Total Nucleic Acid Isolation Kit (Thermo Fisher Scientific, Waltham, USA) according to the manufacturers’ instructions. The cfDNA quantity was assessed with the dsDNA HS assay kit by the Qubit 2.0 Fluorometer (Thermo Fisher Scientific). cfDNA quality was assessed with the Agilent High Sensitivity D1000 ScreenTape System (Agilent Technologies, Santa Clara, USA). Only cfDNA samples with a clear fragment size peak between 140–200 bp (Supplementary Fig. 1) were considered for analysis.

Tissue biopsies were obtained at the time of first diagnosis and inspected through examination of hematoxylin and eosin-stained slides by a thoracic pathologist. For DNA extraction, 4–5 FFPE tissue sections of 10 µm thickness were cut and deparaffinized using Xylol. DNA extraction from tissue was performed using the column-based RecoverAll Extraction Kit (Thermo Fisher Scientific) according to the manufacturer’s instructions. DNA quantity was assessed with the dsDNA HS assay kit by the Qubit 2.0 Fluorometer (Thermo Fisher Scientific).

### NGS library preparation

For plasma samples, NGS libraries were prepared from 2.5 to 105.5 ng of cfDNA following the HeliXmoker, HeliXgyn, and HeliXafe workflows (patented by The Bioscience Institute), based on the Oncomine™ Lung cfDNA Assay v1, the Oncomine™ Breast cfDNA Research Assay v2, and the Oncomine™ Pan-Cancer Cell-Free Assay (Thermo Fisher Scientific), respectively. Only six samples were selected for a broader mutational analysis using the HeliXafe protocol, based on the cfDNA concentration and quality, providing that these six samples matched the required minimum input for a second round of library preparation. Our general library preparation protocol was based on a two-cycle multiplex touch-down PCR reaction with a temperature range from 64 °C to 58 °C, which allowed to amplify target regions and introduce unique molecular identifiers. The resulting tagged amplicons of around 100–140 bp length were then cleaned up using Agencourt AMPure XP (Beckman Coulter, Brea, USA) at a bead to sample ratio of 1.5× and purified products were eluted in 24 μl low TE buffer. A second round of PCR (18 cycles) was performed in a total volume of 50 μl to amplify the purified amplicons and introduce Ion Torrent™ Tag-Sequencing adapters containing sample-specific barcodes. The resulting library of target DNA fragments was purified by performing a two-step cleanup using Agencourt AMPure XP (Beckman Coulter) at a bead to sample ratio of 1.15× and 1.0×, respectively. The purified libraries were then diluted 1:1000 and quantified by qPCR using the Ion Universal Quantitation Kit (Thermo Fisher Scientific). The quantified stock libraries were then diluted to 100 pM for downstream template preparation.

For NGS library preparation from tissue samples 5–40 ng of DNA was used, depending on availability of input material. Libraries were prepared according to protocol (Oncomine™ Solid Tumor Assay, Oncomine™ Focus Assay, Oncomine™ Comprehensive v3 Assay were used (Supplementary Table 1)). The resulting libraries were purified using Agencourt AMPure XP (Beckman Coulter). Libraries were quantified by qPCR using the Ion Universal Quantitation Kit (Thermo Fisher Scientific), diluted to 50 pM and batched according to the manufacturer’s instructions.

### Sequencing

NGS libraries were sequenced on an Ion S5™ instrument (Thermo Fisher Scientific) using semiconductor sequencing technology. Briefly, sequencing runs were planned on the Torrent Suite Software™ v5.8, libraries were pooled and loaded on an Ion 540™ chip using the Ion Chef™ instrument (Thermo Fisher Scientific). The loaded chip was then sequenced using 500 flows. Raw data were processed automatically on the Torrent Server™ and aligned to the reference hg19 genome. QC was performed manually for each sample based on the following metrics; number of reads per sample > 2,500,000 (for Oncomine™ Lung cfDNA Assay libraries), >4,000,000 (for Oncomine™ Breast cfDNA Research Assay v2 libraries) >15,000,000 (for Oncomine™ Pan-Cancer Cell-Free Assay libraries), on-target reads >90%, read uniformity >90%, median molecular coverage >500×, median read coverage >15,000.

Tissue NGS libraries were sequenced according to the manufacturer’s instructions.

The sequencing data of the QC passing samples were then uploaded in BAM format to the Ion Reporter™ Analysis Server for variant calling and annotation.

### Data analysis

For plasma samples variant calling was performed on Ion Reporter™ (IR) Analysis Software v5.6 using the Oncomine™ TagSeq Breast v2 Liquid Biopsy w2.0, Oncomine™ Lung Liquid Biopsy w1.3, and Oncomine™ TagSeq Pan-Cancer Liquid Biopsy w2.0 workflows. The analysis pipeline also included signal processing, base calling, quality score assignment, adapter trimming, PCR duplicate removal, and control of mapping quality. Coverage metrics for each amplicon was obtained by running the Coverage Analysis Plugin software v5.6.1 (Thermo Fisher Scientific). Identified variants were only considered if the variant had a molecular coverage of at least three, indicating that the variant was detected in three independent template molecules. Finally, all candidate mutations were manually reviewed using the Integrative Genomics Viewer^37^.

For tissue samples, the default analysis pipeline in IR (Oncomine™ Solid Tumor Assay, Oncomine™ Focus Assay, Oncomine™ Comprehensive v3) was used.

## Results

### Plasma volume and cfDNA amount define LOD for variant calling

**Fig. 1:**
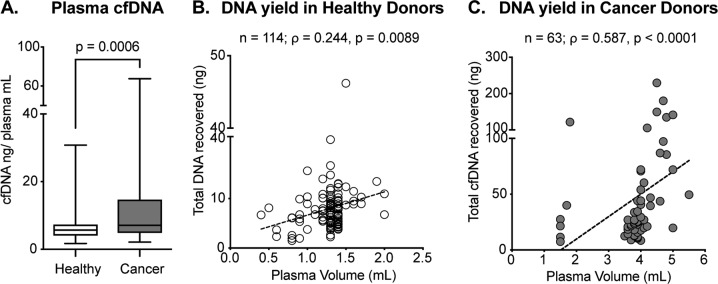
Total cfDNA yield of plasma samples deriving from healthy donors or cancer patients. **a** cfDNA concentration in plasma of healthy individuals compared to cancer patients (Mann–Whitney *p* = 0.0006). Median, interquartile range, and minimum/maximum are shown in the boxplot. **b** Correlation of plasma volume and the total cfDNA output in healthy donors (*n* = 114, Spearman *ρ* = 0.244, *p* = 0.0089). **c** Correlation between the plasma volume and the total cfDNA output in cancer patients (*n* = 63, Spearman *ρ* = 0.587, *p* < 0.0001)

**Fig. 2:**
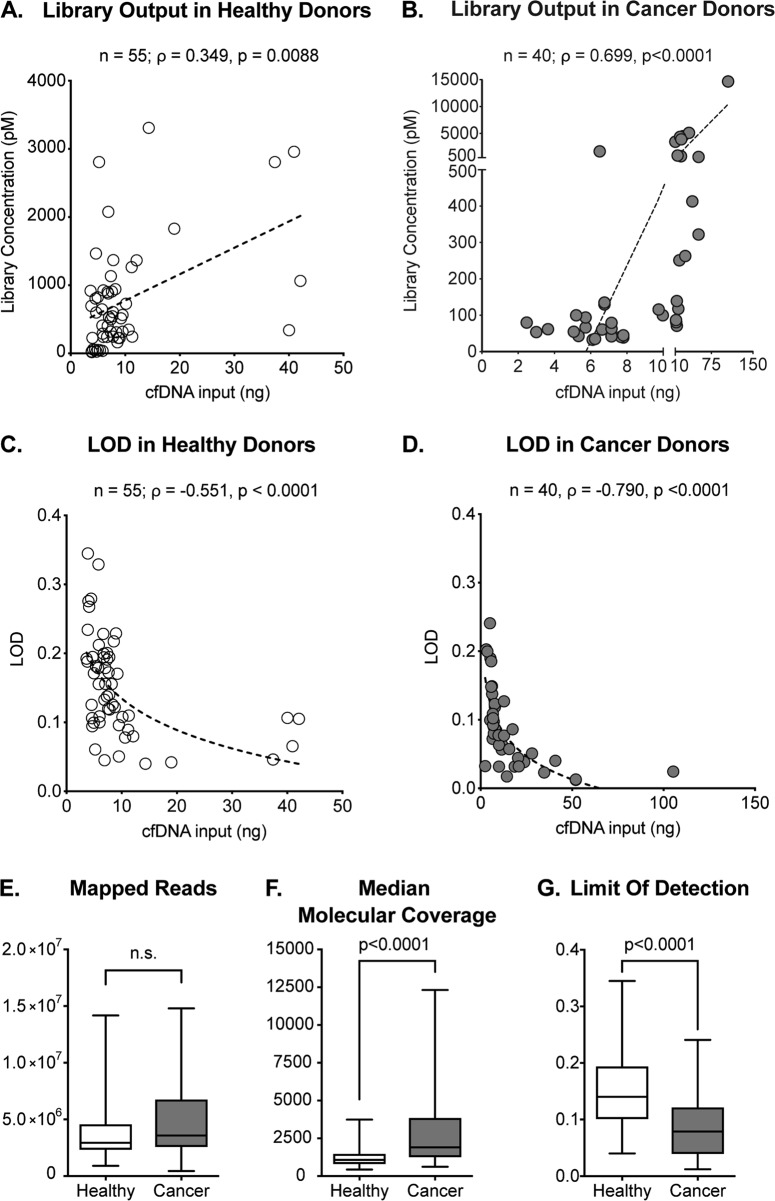
Comparison of pre-analytical variables from healthy and cancer donor samples. **a**, **b** Correlation of library concentration and input of cfDNA in healthy individuals (*n* = 55, Spearman *ρ* = 0.348, *p* = 0.0088) and cancer patients (*n* = 40, Spearman *ρ* = 0.699, *p* < 0.0001). **c**, **d** Correlation of LOD and cfDNA input in healthy (*n* = 55; Spearman *ρ* = −0.551, *p* < 0.0001) and cancer donors (*n* = 40; Spearman *ρ* = −0.790, *p* < 0.0001). **e** Mapped reads of samples deriving from healthy and cancer donors (Mann–Whitney *p* = 0.1422). **f**, **g** Median molecular coverage (Mann–Whitney *p* < 0.0001) and LOD (Mann–Whitney *p* < 0.0001) in healthy and cancer donors. Median, interquartile range, and minimum/maximum are shown in the boxplot

First, we attempted to establish a solid workflow for the extraction of cfDNA from plasma of either healthy individuals or cancer patients. Table 1 summarizes the analyzed cohort characteristics. Peripheral whole blood was collected in commercial vessels containing EDTA or a preservative agent preventing cell lysis and thus the contamination of circulating cfDNA with cellular DNA. After plasma isolation, we extracted cfDNA using a magnetic beads-based kit as described in detail in the Materials and methods section. The amount of plasma available varied between 0.4 and 2.0 ml in healthy individuals and 1.5 and 5.5 ml in cancer patients (Fig. 1b, c, Materials and methods). As previously reported (first in 1977 (ref. ^38^)), total cfDNA concentration in plasma was significantly higher in cancer patients compared to healthy subjects (*p* = 0.0006, Fig. 1a). We characterized the correlation between plasma input and total cfDNA yield in samples collected from healthy donors (*ρ* = 0.244, *p* = 0.0089, shown in Fig. 1b) and cancer patients (*ρ* = 0.587, *p* < 0.0001, shown in Fig. 1c). Next, we processed cfDNA samples from healthy and cancer donors for NGS library preparation and sequencing. NGS library concentration was significantly affected by cfDNA input in both healthy and cancer samples (*ρ* = 0.348, *p* = 0.0088 and *ρ* = 0.699, *p* < 0.0001, respectively, Fig. 2a, b). Notably, as healthy individuals generally present with lower levels of cfDNA compared to cancer patients, limited DNA input was used for library preparation, often below the minimal manufacturers’ recommended amount (i.e. 10 ng). We show that the limit of detection (LOD) of our assay, which indicates the lowest variant allelic frequency that could be reliably detected, is clearly affected by the cfDNA abundance in both healthy individuals and cancer patients (Fig. 2c, d) with an inverse correlation between these two variables. Despite comparable sequencing depth in healthy and cancer donor samples (Fig. 2e), we observed higher molecular coverage in cancer samples (Fig. 2f, *p* < 0.0001) due to higher amount of input cfDNA. For the same reason the LOD was significantly lower in cancer patients (Fig. 2g, *p* < 0.0001). Thus, our data show that the amount of cfDNA has a direct impact on sequencing performance and LOD.

### cfDNA profiling of cancer patients and concordance with tissue

**Fig. 3:**
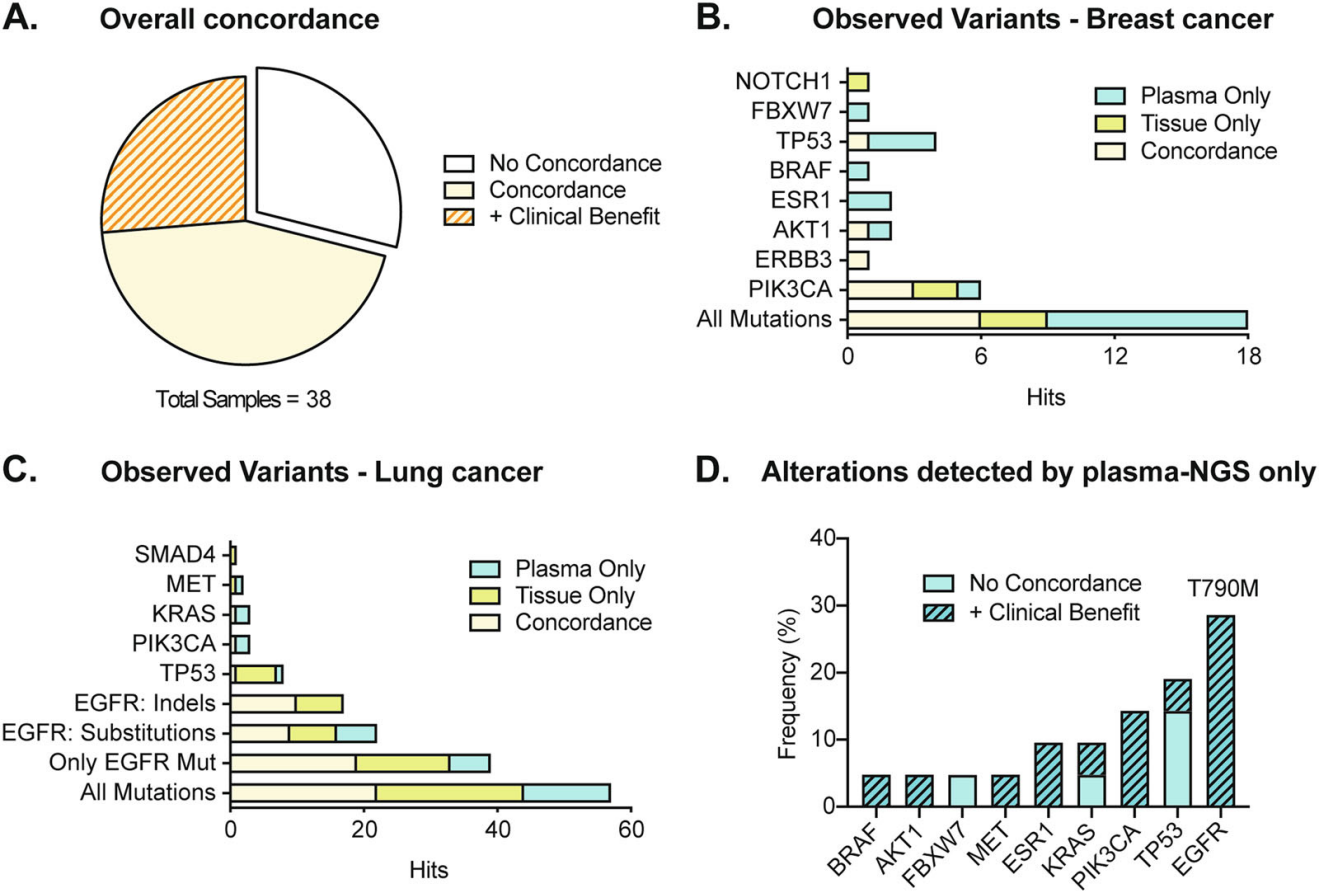
Concordance analysis of liquid and tissue biopsy in cancer patients. **a** Representation of the percentage of overall concordance of matched tissue and liquid biopsy. “+Clinical benefit” refers to additional clinically relevant mutations that were detected through NGS analysis of liquid biopsy and not tissue biopsy (see “plasma only” in the next sections). No concordance was observed in 29% of the samples, whereas out of 71% concordant samples 26% carried additional clinically relevant mutations detected by plasma only (+ Clinical Benefit). **b**, **c** Number of observed variants for breast (**b**) and lung (**c**) cancer samples. Only clinically relevant variants covered by both tissue and plasma NGS panels were considered for the analysis. **d** Distribution of gene alterations detected by NGS analysis of plasma and not detected in tissue (total *n* = 24). Among the clinically relevant mutations that were detected through NGS analysis of liquid biopsy and not tissue biopsy, the most frequent (32%) is T790M in EGFR. Mutations found by plasma alone were subdivided in the “+ Clinical Benefit” category if they were part of additionally clinically relevant mutations detected by plasma alone in samples showing overlap in tissue and plasma mutational profiles (i.e. concordance for oncogenic drivers). The “No Concordance” category indicates mutations detected in samples showing no overlap in tissue and plasma mutational profiles

Previous studies demonstrated the high analytical sensitivity of using molecular barcodes, also referred to as unique molecular identifiers (UMIs^23,24,39^) for NGS. Here we attempted to investigate the concordance, in terms of corresponding detected variants, between circulating cfDNA and matched tissue from primary tumor or metastasis of the same patient. To this end, we analyzed cfDNA obtained from eight breast cancer patients using the HeliXgyn protocol (developed by the Bioscience Institute and based on the Oncomine™ cfDNA Breast v2 Assay) and 30 non-small cell lung cancer (NSCLC) patients using the HeliXmoker protocol (developed by the Bioscience Institute and based on the Oncomine™ cfDNA Lung Assay) and sequentially compared it with the results obtained by sequencing tissue using a suitable Oncomine™ Assay (detailed about used gene panels in Supplementary Table 1). We used molecular barcoded sequencing (Tag Sequencing barcodes) to profile our liquid biopsy samples. As the target regions of the panels used for cfDNA and tissue profiling were not fully overlapping, we focused only on clinically relevant mutations covered by both panels (Supplementary Table 1). Our data highlight (Fig. 3a) a substantial level of concordance (71%) between cfDNA and tissue mutational profiles of matched samples. This suggests that cfDNA analysis reliably mimics tissue genomic features. Furthermore, additional clinically relevant mutations were detected by liquid biopsy in 26% of the samples showing a concordant result (Fig. 3a, “plus Clinical Benefit”). The most frequently observed mutations occurred within the coding region of PIK3CA (6 out of 18 mutations detected) for breast cancer (Fig. 3b) and EGFR (40 out of 57 mutations detected) for NSCLC specimens (Fig. 3c). All mutations detected are summarized in a concordance matrix (Supplementary Fig. 2A, B for breast and lung cancer samples, respectively). In breast cancer samples, we found concordance for mutations detected in PIK3CA, AKT1, and ERBB3, whereas mutations in TP53, ESR1, and BRAF were more often detected by plasma alone (Fig. 3b). In lung cancer samples, deletions in the EGFR coding regions were more often detected only by tissue, whereas for substitution in EGFR we observed a more prevalent fraction detected only by plasma (Fig. 3c). The time interval between tissue and blood collection ranged from 0 to 70 months with intervening treatment, suggesting that tumor evolution and not only tumor heterogeneity could be the underlying reason for incongruence between tissue and liquid biopsy analysis. We analyzed the effect of time occurring between tissue biopsy and blood collection on concordance (shown in Supplementary Fig. 2C). We observed a trend of decreased time interval between tissue and liquid biopsy for concordant samples, however, without reaching statistical significance (*p* = 0.4325). Among mutations detected by plasma only, the EGFR T790M resistance mutation was the most frequent (32% of all mutations detected by plasma and not by tissue NGS analysis, Fig. 3d). This mutation was likely not detected in the initial tissue biopsy because it is known to emerge during therapy as a resistance mechanism against tyrosine-kinase inhibitor treatment of EGFR-mutated tumors. These data confirm the effectiveness of our testing strategy and highlight the clinical value of using liquid biopsy as a complementary tool to tissue biopsy for monitoring tumor evolution during treatment.

### cfDNA profiling of healthy individuals

**Fig. 4:**
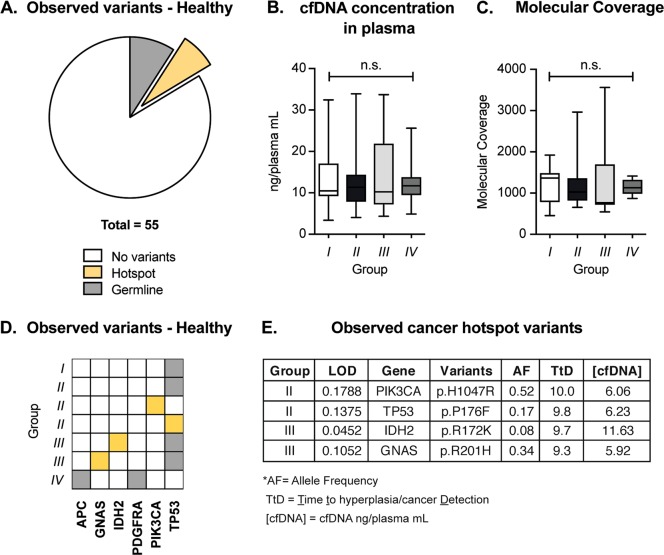
Genetic alterations detected in the cfDNA of healthy individuals. **a** No genetic alteration was detected in 84% of the assayed samples; however, we detected six germline and four hotspot variants in seven different samples. **b**, **c** Pre-analytical variables as cfDNA concentration in plasma (**b**) and median molecular coverage (**c**) in the four groups of healthy donors (Kruskal–Wallis *p* = 0.9223 and *p* = 0.7721, respectively). Group I: healthy at follow-up time; group II: benign breast condition at follow-up time; group III: breast cancer at follow-up time; group IV: a solid tumor other than breast cancer at follow-up time. Median, interquartile range, and minimum/maximum are shown in the boxplot. **d** Mutational matrix indicating the variants detected in healthy individuals belonging to the four groups. Each line represents a patient. Yellow squares represent hotspot variants; gray squares represent germline variants. **e** Table summarizing the hotspot variants detected in healthy individuals. LOD limit of detection, AF allele frequency; TtD Time to hyperplasia/cancer Detection, [cfDNA] cfDNA concentration in plasma (cfDNA ng/plasma ml)

Finally, we attempted to profile the cfDNA of individuals that were healthy (as above defined) at the time of blood collection. Our patient cohort comprised *n* = 106 women that underwent a control screening mammography test and had been followed-up regularly for up to 10 years later (Table 1). Mammography screening and blood collection were performed concurrently. For this study, we divided the healthy individuals into four groups based on clinical status at follow-up (Table 1). Individuals belonging to group I (*n* = 25) did not develop any breast cancer or other malignancies during follow-up time. In group II individuals (*n* = 52) experienced fibrocystic breast changes such as fibroadenoma and hyperplasia during follow-up time, while in group III (*n* = 15) they developed breast cancer. Donors allocated to group IV (*n* = 14) developed a solid tumor other than breast cancer (specifically: laryngeal squamous cell carcinoma, glioblastoma multiforme, basal cell carcinoma, and thyroid cancer). The average follow-up time was 8.5 years and did not differ significantly between the four groups described (Supplementary Fig. 3, Table 1). As reported in the first section of our results, we successfully achieved cfDNA extraction from all plasma samples, with values ranging from 1.7 to 30.8 ng/ml of cfDNA (Fig. 1a). Based on recovery rate and quality of cfDNA (described in Supplementary Fig. 1 and Material and methods), we selected 55 samples for downstream NGS analysis (group I = 12/25; group II = 23/52; group III = 11/15; group IV = 9/14; total = 55/106). We processed the selected samples using the HeliXgyn workflow and we selected six samples for a broader mutational analysis using the HeliXafe protocol (based on the Oncomine™ cfDNA Pan-Cancer Assay). The turnaround time from start of plasma processing to data analysis was on average six working days for these 55 samples, confirming that we have established a fast workflow (Supplementary Fig. 2D). The results of the molecular profiling are summarized in Fig. 4. No genetic alterations were found in the cfDNA of most healthy individuals (84%) (Fig. 4a). Among the four groups of healthy individuals with different outcomes at follow-up, no significant difference was observed in terms of pre-analytical variables, including cfDNA concentration in plasma or achieved molecular coverage (Fig. 4b, c). In 7 of the 55 cases analyzed, we detected clinically relevant gene mutations, specifically six known germline variants observed at allelic frequencies above 40% and four known cancer hotspot mutations (Fig. 4d, e). In conclusion, our results provide evidence that genetic alterations related to cancer occurrence can be detected in healthy individuals by analyzing cfDNA.

## Discussion

Liquid biopsy has recently gained substantial attention in the field of cancer diagnostics. Ambitious efforts are currently placed towards the implementation of liquid biopsy as an early cancer detection method (i.e. before cancer-related symptoms occur) and ctDNA mutation analysis has already been reported in early-stage tumors^26–28,40^. Early diagnosis possibly equals to a better disease outcome; however, large-scale validation studies are required to better understand the full potential and the limitations of this application of liquid biopsy^36^. The screening of pre-cancerous lesions in asymptomatic individuals is hindered by several challenges. Namely, the number of mutant ctDNA molecules present in plasma is mostly proportional to tumor burden^9^, rendering detection particularly problematic in patients with localized cancer and asymptomatic individuals. Another challenge is represented by the lack of knowledge regarding the molecular basis of tumor initiation. Several studies have reported the detection of somatic mutations and related clonal expansion in healthy tissue^30–33^ associated with age and tissue proliferative rate^41^. Some of these mutations were shown to increase the risk of developing cancer^42,43^. The Pre-Cancer Genome Atlas^44^ will significantly improve our understanding of the role of pre-cancerous lesions in early stages of tumor formation, improving the specificity of early detection screening. At present liquid biopsy is mainly used in advanced cancer patients; however, the Circulating Cell-free Genome Atlas (CCGA) study and the development of early screening methods such as CancerSEEK^27^ are opening the way for cfDNA testing in healthy individuals and early-stage tumor patients. Our work aimed to contribute to this field by investigating the technical feasibility of using liquid biopsy for screening healthy individuals. Our cohort comprises 177 individuals, out of which 114 were clinically healthy and 63 were diagnosed with breast or lung cancer at time of blood collection. Because of the design of our study, which included patients undergoing routine mammography screening and followed-up for breast cancer insurgence, all healthy volunteers analyzed were women. As expected, cfDNA concentration was significantly lower in plasma from healthy individuals compared to cancer patients (Fig. 1a), with cfDNA concentrations ranging from 1 to 16.8 ng ml^−1^ for healthy individuals (with the exception of one sample which had a concentration of 30.8 ng ml^−1^ in plasma), consistently with previously published results^34,35^. The patient presenting 30.8 ng ml^−1^ in plasma belonged to group II. We did not detect any mutation for this sample nor found any sign of genomic contamination. Raised cfDNA concentrations have been observed in healthy donors under several physiological conditions (as physical exercise^45^ or infection^46^). To overcome the challenges associated with low input material as well as enabling the detection of low-frequency mutations, we have implemented molecular barcoding^13,23,24,39^ (reviewed in ref. ^47^) in our sample processing workflow. We have confirmed the reliability and accuracy of our method by matched genomic analysis of tissue and plasma samples in cancer patients (concordance of 71% for our cohort of breast and lung cancer patients; Fig. 3a). These results are in line with previous studies reporting sensitivity between 65% and 98%^48–51^ (reviewed in ref. ^7^). We did not observe perfect concordance possibly due to tumor heterogeneity and evolution under treatment pressure (Supplementary Fig. 2C). We then used this method to screen for signs of genomic instability in healthy donors. We could successfully isolate cfDNA and produce functional NGS libraries from as little as 0.9 ml of plasma; however, we recovered material of adequate quality to undergo NGS library preparation only for 55 out of 114 patients. Moreover, we observed higher LOD (Fig. 2g) in healthy donors compared to cancer patients due to higher cfDNA input in cancer patients. The availability of lower amounts of plasma for cfDNA isolation in healthy donors (0.4 and 2.0 ml in healthy individuals and 1.5 and 5.5 ml in cancer patients) is a drawback of this study. As healthy individuals present with lower levels of cfDNA compared to cancer patients (Fig. 1a), we recommend using higher volumes of plasma for cfDNA analysis from healthy donors. Importantly, this would allow for the detection of variants present at low allelic frequencies, which could be particularly relevant for discovering the presence of early genomic changes (as shown by the four cancer hotspot mutations we identified, Fig. 4d). Through our analysis, we detected genetic alterations in 7 out of 55 subjects with evaluable cfDNA that were considered clinically healthy at the time of liquid biopsy. Among these mutations, we found six germline variants and four cancer hotspot mutations. The observation of germline variants is a byproduct of our cfDNA analysis. Interestingly, many germline variants detected in our study are mutations in the coding region of TP53 that have been consistently reported to correlate with genomic instability and increased cancer risk^52–55^. Those patients might be recommended to have genetic counseling and upon the decision of a trained certified geneticist to access early prevention programs. The four cancer hotspot mutations detected are recurrent genetic alterations, clinically classified as pathogenic or likely pathogenic. Previous studies have identified mutations in saliva and plasma of individuals up to 2 years before tumor insurgence^56,57^. We detected cancer hotspot variants in individuals that were diagnosed with a benign breast condition (group II) or breast cancer (group III) up to 10 years later and at allelic frequencies ranging from 0.08% to 0.52% (Fig. 4d). Furthermore, the detected hotspot mutations have been associated with breast cancer as well as non-neoplastic proliferation of tissue by several studies^58–69^. Observing these mutations in the cfDNA of healthy donors might be considered as indirect evidence of genomic instability, as was shown for the PIK3CA p.H1047R variant^62^. However, it was also observed that pathogenic TP53 mutations can be detected in the cfDNA of healthy controls^70^ with no correlation to tumor insurgence. Therefore, the interpretation of these findings warrants caution and needs to be carefully considered before drawing any conclusion. Additional extensive prospective studies with long follow-up time and available tissue specimens for individuals who develop cancer will be required to address the specificity and sensitivity of liquid biopsy as a tool for early cancer detection. In conclusion, with this work we have established a rapid and reliable workflow that allowed us to interrogate cfDNA from healthy individuals to study genomic alterations with a limit of detection as low as 0.08% allelic frequency. The interrogation of cfDNA from the blood of healthy individuals could prove to be a prospective tool to detect signs of genomic instability and to better understand early events in tumor formation.

## Supplementary information

Supplementary Table 1.

Supplementary Figure 1.

Supplementary Figure 2.

Supplementary Figure 3.

Supplementary figure legends.
