# Supplementary material for: Cell-free DNA analysis in healthy individuals by next-generation sequencing: a proof of concept and technical validation study
Source: Cell Death Dis. 2019 Jul 11;10(7):534. doi: 10.1038/s41419-019-1770-3 (PMC6624284; doi:10.1038/s41419-019-1770-3)
Supplement: Supplementary file 1 — Supplementary Table 1. [file 41419_2019_1770_MOESM1_ESM.docx]

**Supplementary Table 1: Gene content of the Oncomine™ NGS panels for cfDNA and tissue analysis.**

| **Breast**  **cfDNA v2** | **Lung**  **cfDNA** | **Pancancer cfDNA** | **Solid Tumour DNA Kit** | **Focus DNA Assay** |
| --- | --- | --- | --- | --- |
| AKT1 | ALK | AKT1 | AKT1 | AKT1 |
| EGFR | BRAF | ALK | ALK | ALK |
| ERBB2 | EGFR | APC | BRAF | APC |
| ERBB3 | ERBB2 | AR | CTNNB1 | AR |
| ESR1 | KRAS | ARAF | DDR2 | BIRC2 |
| FBXW7 | MAP2K1 | BRAF | EGFR | BRAF |
| KRAS | MET | CCND1 | ERBB2 | BRCA1 |
| PIK3CA | NRAS | CCND2 | ERBB4 | CCND1 |
| SF3B1 | PIK3CA | CCND3 | FBXW7 | CDK4 |
| TP53 | ROS1 | CDK4 | FGFR1 | CDK6 |
|  | TP53 | CDK6 | FGFR2 | CTNNB1 |
|  |  | CHEK2 | FGFR3 | DCUN1D1 |
|  |  | CTNNB1 | KRAS | DDR2 |
|  |  | DDR2 | MAP2K1 | EGFR |
|  |  | EGFR | MET | ERBB2 |
|  |  | ERBB2 | NOTCH1 | ERBB3 |
|  |  | ERBB3 | NRAS | ERBB4 |
|  |  | ESR1 | PIK3CA | ESR1 |
|  |  | FBXW7 | PTEN | FGFR1 |
|  |  | FGFR1 | SMAD4 | FGFR2 |
|  |  | FGFR2 | STK11 | FGFR3 |
|  |  | FGFR3 | TP53 | FGFR4 |
|  |  | FGFR4 |  | GNA11 |
|  |  | FLT3 |  | GNAQ |
|  |  | GNA11 |  | HRAS |
|  |  | GNAQ |  | IDH1 |
|  |  | GNAS |  | IDH2 |
|  |  | HRAS |  | JAK1 |
|  |  | IDH1 |  | JAK2 |
|  |  | IDH2 |  | JAK3 |
|  |  | KIT |  | KIT |
|  |  | KRAS |  | KRAS |
|  |  | MAP2K1 |  | MAP2K1 |
|  |  | MAP2K2 |  | MAP2K2 |
|  |  | MET |  | MED12 |
|  |  | MTOR |  | MET |
|  |  | MYC |  | MTOR |
|  |  | NRAS |  | MYC |
|  |  | NTRK1 |  | MYCN |
|  |  | NTRK3 |  | NF1 |
|  |  | PDGFRA |  | NRAS |
|  |  | PIK3CA |  | PDGFRA |
|  |  | PTEN |  | PIK3CA |
|  |  | RAF1 |  | RAF1 |
|  |  | RET |  | RET |
|  |  | ROS1 |  | ROS1 |
|  |  | SF3B1 |  | SMO |
|  |  | SMAD4 |  |  |
|  |  | SMO |  |  |
|  |  | TP53 |  |  |
