# Supplementary figures and images for: Cell-free DNA analysis in healthy individuals by next-generation sequencing: a proof of concept and technical validation study
Source: Cell Death Dis. 2019 Jul 11;10(7):534. doi: 10.1038/s41419-019-1770-3 (PMC6624284; doi:10.1038/s41419-019-1770-3)

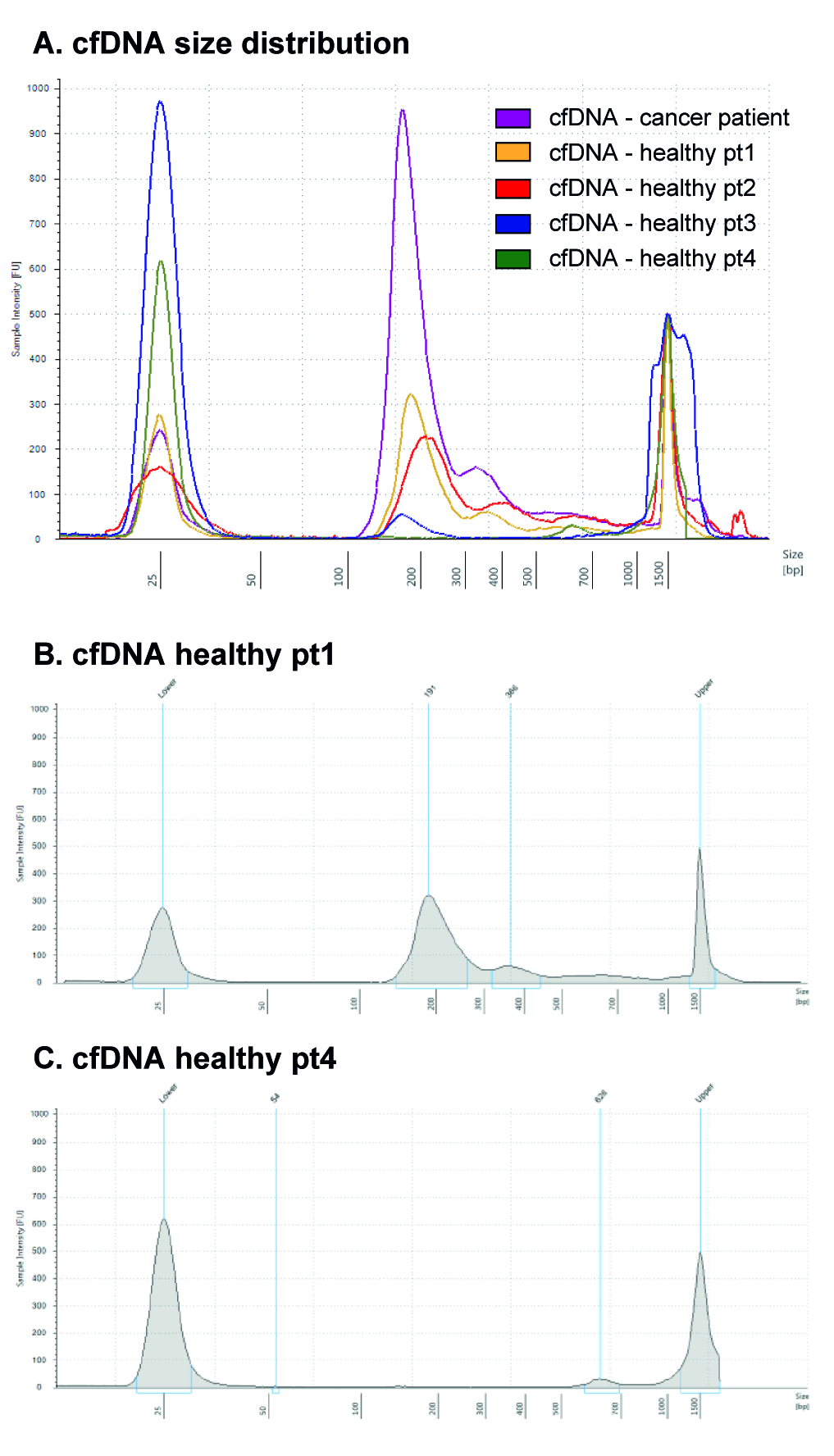

Supplement: Supplementary file 2 — Supplementary Figure 1. [file 41419_2019_1770_MOESM2_ESM.tif]

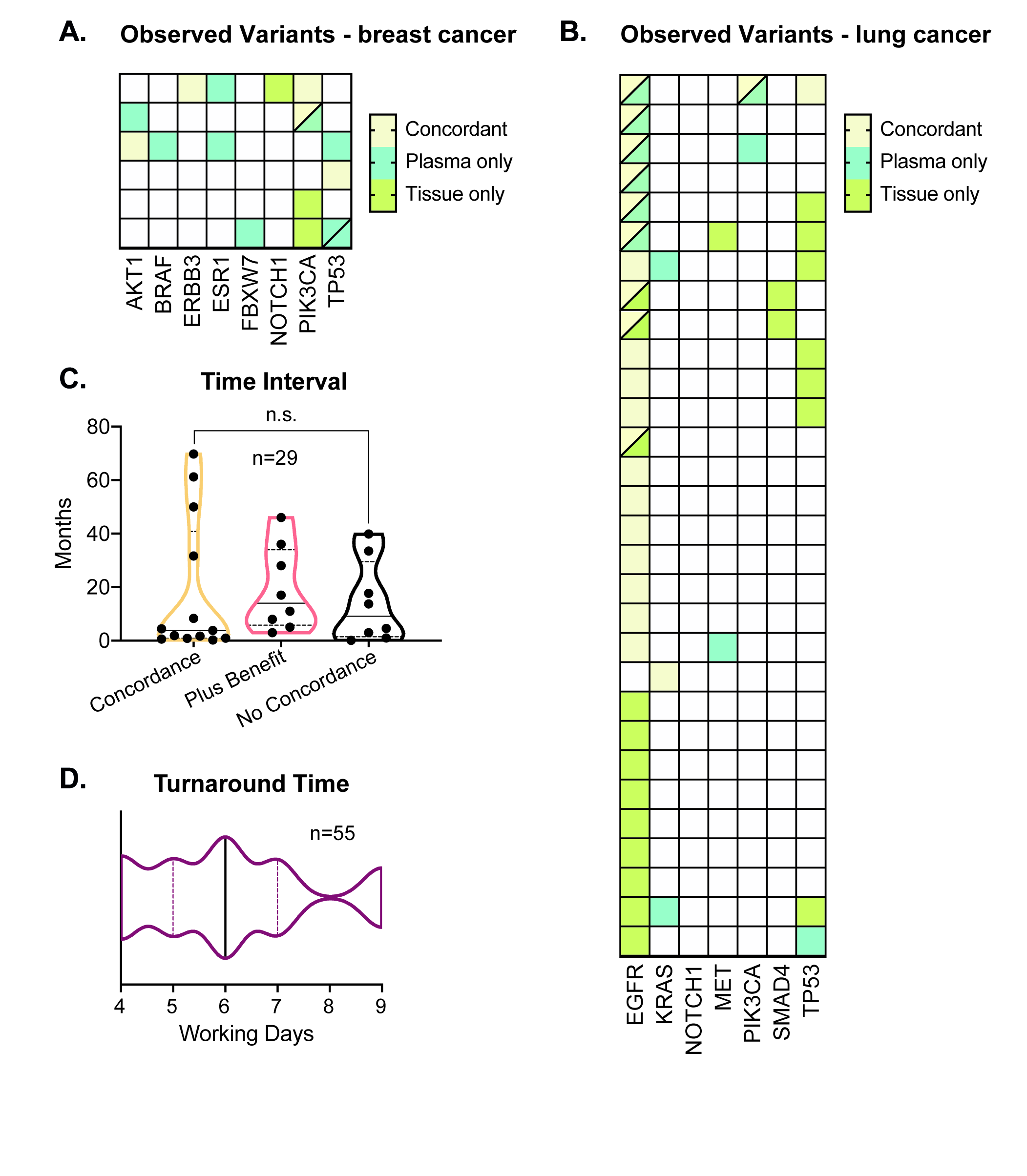

Supplement: Supplementary file 3 — Supplementary Figure 2. [file 41419_2019_1770_MOESM3_ESM.tif]

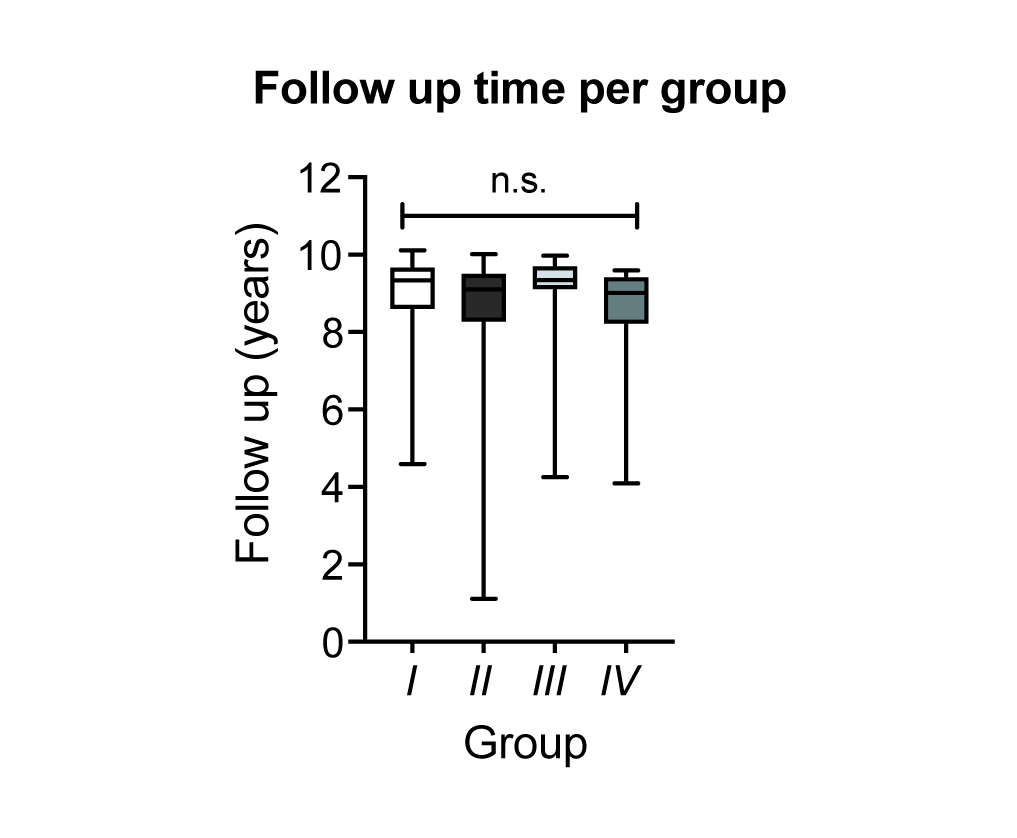

Supplement: Supplementary file 4 — Supplementary Figure 3. [file 41419_2019_1770_MOESM4_ESM.tif]
