# Supplementary material for: Cell-free DNA analysis in healthy individuals by next-generation sequencing: a proof of concept and technical validation study
Source: Cell Death Dis. 2019 Jul 11;10(7):534. doi: 10.1038/s41419-019-1770-3 (PMC6624284; doi:10.1038/s41419-019-1770-3)
Supplement: Supplementary file 5 — Supplementary figure legends. [file 41419_2019_1770_MOESM5_ESM.docx]

**Supplementary Figure 1.** **cfDNA size distribution in healthy individuals and cancer patients.** **A.** cfDNA size distribution of a cancer patient (purple line) compared to healthy donors (yellow, red, blue, green line). **B.** cfDNA profile of a healthy donor (cfDNA healthy 1, red line in A) with clear fragment size peaks at 191 bp. **C.** cfDNA profile of a healthy donor (cfDNA healthy 4, green line in A) with no fragment size peaks at the expected range for cfDNA. Only cfDNA samples with a clear fragment size peak between 140 – 200 bp were selected for NGS analysis.

**Supplementary Figure 2. Concordance analysis of matched plasma and tissue samples from breast and lung cancer patients.** **A-B.** Concordance matrix of breast (A) and lung (B) cancer samples, each line represents a patient. Blue squares represent variants detected by plasma only, green squares represent variants detected by tissue only, yellow squares represent variants detected by both plasma and tissue. A split squares represents 2 variants detected in the same gene for the same sample. Wild-type patients were not plotted (2 breast cancer patients were wild-type for all genes analyzed). **C.** Violin plot showing the effect of time between blood and tissue collection on concordance (n=29, Kruskal-Wallis p=0.4325; values missing for 9 out of 38 samples, median for concordance group=3.8 months, median for plus benefit group=12 months, median for no concordance group=9 months). Median and minimum/maximum are represented in the plot. **D.** Turnaround time of our liquid biopsy workflow. Working days were counted from start of sample processing to final molecular report for 55 samples (from healthy donors, shown in Figure 4). Average/median = 6 working days. Solid line shows median, dotted line marks quartiles.

**Supplementary Figure 3. Follow-up time did not significantly differ between the four groups of healthy donors (Kruskal-Wallis p=0.1971).** Group I: healthy at follow-up time; group II: benign breast condition at follow-up time; group III: breast cancer at follow-up time; group IV: a solid tumor other than breast cancer at follow-up time. Median, interquartile range and minimum/maximum are shown in the boxplot.
